# Supplementary material for: Food-Based Dietary Guidelines for Infants in Latin America and the Caribbean: A Systematic Review
Source: Nutrients. 2024 Apr 21;16(8):1233. doi: 10.3390/nu16081233 (PMC11053862; doi:10.3390/nu16081233)
Supplement: Supplementary file 1 [file nutrients-16-01233-s001.zip › Supplementary Material Table S1.pdf]

Supplementary Material Table S1: Original Score Scale based on Agree II for Overall assessment of FBDG.

Agree II formula:

Example:

If 4 appraisers give the following scores for Domain 1 (Scope & Purpose):

	Item 1	Item 2	Item 3	**Total**
Appraiser 1	5	6	6	**17**
Appraiser 2	6	6	7	**19**
Appraiser 3	2	4	3	**9**
Appraiser 4	3	3	2	**8**
**Total**	**16**	**19**	**18**	**53**

Maximum possible score = 7 (strongly agree) x 3 (items) x 4 (appraisers) = 84
Minimum possible score = 1 (strongly disagree) x 3 (items) x 4 (appraisers) = 12

The scaled domain score will be:

$$\frac{\text{Obtained score} - \text{Minimum possible score}}{\text{Maximum possible score} - \text{Minimum possible score}}$$

$$\frac{53 - 12}{84 - 12} \times 100 = \frac{41}{72} \times 100 = 0.5694 \times 100 = 57\ \%$$

If items are not included, appropriate modifications to the calculations of maximum and minimum possible scores are required.

For each domain it can rate 100% percent as maximum, so we considered 600% as maximum rate for Overall Assessment and added all the domain scores for each Guide, resulting in a score that variated from 0% to 600%, with ranges of 85% (for the 7 items of Likert Scale, 600/7= 85). This way, the overall score classified the Guide in one of the 7 categories. All of the calculations were made in Excel.
