# Supplementary material for: Food-Based Dietary Guidelines for Infants in Latin America and the Caribbean: A Systematic Review
Source: Nutrients. 2024 Apr 21;16(8):1233. doi: 10.3390/nu16081233 (PMC11053862; doi:10.3390/nu16081233)
Supplement: Supplementary file 1 [file nutrients-16-01233-s001.zip › Supplementary Material Table S2.pdf]

Supplementary Material Table S2: LIST OF RESEARCHED ASSOCIATIONS AND INSTITUTIONS (GRAY LITERATURE)

Institution	Link
Ministerio de Salud (Gobierno de Chile)	https://www.minsal.cl/politicas-publicas-en-alimentacion-y-nutricion/
Guao (biblioteca del gobierno de Venezuela)	https://www.guao.org/biblioteca/alimentacion_del_nino_y_la_nina_preescolar_y_escolar
INAN (Instituto Nacional de Alimentación y Nutrición)	https://www.inan.gov.py/site/
Ministerio de Salud Peruano	https://www.gob.pe/institucion/minsa/informes-publicaciones/314037-guias-alimentarias-para-la-poblacion-peruana
Ministerio de Salud y Deportes de Bolivia	https://www.minsalud.gob.bo/
Instituto Colombiano de Bienestar Familiar ICBF	https://www.icbf.gov.co/guias-alimentarias-basadas-en-alimentos-para-mujeres-gestantes-madres-en-periodo-de-lactancia-y-2
Ministerio de Desarrollo Social del Uruguay	https://www.gub.uy/buscar?search_api_fulltext=u%C3%ADa+de+alimentaci%C3%B3n+complementaria+para+ni%C3%B1os+de+entre+6+y+24
Secretaria de Salud (Gobierno de Mexico)	https://www.gob.mx/promosalud/documentos/guias-alimentarias-para-la-poblacion-mexicana?state=published
Ministerio de Salud Pública (Gobierno de República Dominicana)	https://repositorio.msp.gob.do/handle/123456789/1249
Instituto de Nutrición de	http://www.sintet.net/biblioteca/guia-alimentaria-para-poblacion-guatemalteca-menor-de-2-anos

Supplementary Material Table S2: LIST OF RESEARCHED ASSOCIATIONS AND INSTITUTIONS (GRAY LITERATURE)

Centro América y Panamá (INCAP)/Ministerio de Salud Pública y Asistencia Social de Guatemala	
Departamento de Salud Nutricional del Ministerio de Salud (MINSA) de la República de Panamá	https://www.fao.org/3/CA1017ES/ca1017es.pdf
Ministerio de Salud y Ambiente de la Nación (Gobierno de Argentina)	https://www.minsalud.gob.bo/images/Libros/DGPS/PDS/p345_g_dgps_uan_GUIA_ALIMENTARIA_PARA_LA_MUJER_DURANTE_EL_PERIODO_DE_EMBARAZO_Y_LACTANCIA.pdf
Ministério da Saúde (Governo Federal do Brasil)	https://www.gov.br/saude/pt-br/assuntos/saude-brasil/eu-quero-me-alimentar-melhor/Documentos/pdf/guia-alimentar-para-criancas-brasileiras-menores-de-2-anos.pdf/view
Ministerio de Salud Pública (Gobierno de Ecuador)	https://www.salud.gob.ec/wp-content/uploads/2019/07/4_alimentacion_ni%C3%B1o_menor_2a%C3%B1os.pdf
Ministerie van Volksgezondheid (Republiek Suriname)	https://gov.sr/ministeries/ministerie-van-volksgezondheid/
Ministry of Health Guyana	https://www.health.gov.gy/
Ministry of Health and	https://www.health.gov.bz/

Supplementary Material Table S2: LIST OF RESEARCHED ASSOCIATIONS AND INSTITUTIONS (GRAY LITERATURE)

Wellness Belize	
Ministerio de Salud (Gobierno de Costa Rica)	https://www.ministeriodesalud.go.cr/
Ministerio de Salud (Gobierno de El Salvador)	https://www.salud.gob.sv/
Secretaria de Salud (Gobierno de la República de Honduras)	https://www.salud.gob.hn/sshome/index.php
Ministerio de Salud (Gobierno de Reconciliación y Unidad Nacional de Nicaragua)	https://www.minsa.gob.ni/
Ministry of Health, Wellness and the Enviroment (Government of Antigua e Barbuda)	https://health.gov.ag/#
Ministry of Health (The Government of The Bahamas)	https://www.bahamas.gov.bs/health/
Ministry of Health and Wellness (Government of Barbados)	https://www.health.gov.bb/
Ministerio de Salud Pública	https://salud.msp.gob.cu/?s=guia+alimentaria+para+ni%C3%B1os

Supplementary Material Table S2: LIST OF RESEARCHED ASSOCIATIONS AND INSTITUTIONS (GRAY LITERATURE)

(República de Cuba)	
Ministry of Health, Wellness and Social Services (Government of Dominica)	https://dominica.gov.dm/ministries/health-wellness-and-social-services
Ministerio de Sanidad (Ayuntamento de Granada)	https://www.granada.org/
Ministère de la Santé Publique et de la Population(République d´Haiti)	https://www.mspp.gouv.ht/
Ministry of Health and Wellness (Government of Jamaica)	https://www.moh.gov.jm/
Ministry of Health, Wellness and Elderly Affairs (Government of Saint Lucia)	https://health.govt.lc/
Ministry of Health and Social Security (Government os Saint Kitti and Nevis)	https://www.gov.kn/
Ministry of Health (Government of the Republic of Trinidad	https://health.gov.tt/

Supplementary Material Table S2: LIST OF RESEARCHED ASSOCIATIONS AND INSTITUTIONS (GRAY LITERATURE)

and Tobago)	

REFERENCES OF ACESSED PORTALS

FAO Food-Based Dietary Guidelines Available online: http://www.fao.org/nutrition/education/food-dietary-guidelines/home/en/ (accessed on 25 October 2023).

Guías Alimentarias menores 2 años Available online: https://www.icbf.gov.co/nutricion/guias-alimentarias-menores-2-anos (accessed on 25 October 2023).

Banco de Recursos de Comunicación Del Ministerio de Salud de La Nación | Guía de Alimentación Infantil Available online: https://bancos.salud.gob.ar/recurso/guia-de-alimentacion-infantil (accessed on 25 October 2023).

Políticas Públicas En Alimentación y Nutrición Available online: https://www.minsal.cl/politicas-publicas-en-alimentacion-y-nutricion/ (accessed on 25 October 2023).

Alimentación Del Niño y La Niña Preescolar y Escolar | Guao Available online: https://www.guao.org/biblioteca/alimentacion_del_nino_y_la_nina_preescolar_y_escolar (accessed on 27 October 2023).

INAN – Instituto Nacional de Alimentación y Nutrición.

Guías alimentarias para la población peruana Available online: https://www.gob.pe/institucion/minsa/informes-publicaciones/314037-guias-alimentarias-para-la-poblacion-peruana (accessed on 27 October 2023).

Bolivia (Plurinational State Of) Available online: http://www.fao.org/nutrition/education/food-dietary-guidelines/regions/bolivia-plurinational-state-of/en/ (accessed on 25 October 2023).

Ministerio de Desarrollo Social Guía Alimentaria Para La Población Uruguaya Available online: https://www.gub.uy/buscar?search_api_fulltext=u%C3%ADa+de+alimentaci%C3%B3n+complementaria+para+ni%C3%B1os+de+entre+6+y+24 (accessed on 27 October 2023).

salud, H. de Guias alimentarias para la población mexicana Available online: http://www.gob.mx/promosalud/documentos/guias-alimentarias-para-la-poblacion-mexicana?state=published (accessed on 25 October 2023).
